# Mitochondrial neuronal uncoupling proteins: a target for potential disease-modification in Parkinson's disease

**DOI:** 10.1186/2047-9158-1-3

**Published:** 2012-01-13

**Authors:** Philip WL Ho, Jessica WM Ho, Hui-Fang Liu, Danny HF So, Zero HM Tse, Koon-Ho Chan, David B Ramsden, Shu-Leong Ho

**Affiliations:** 1 Division of Neurology, Department of Medicine, University of Hong Kong, Pokfulam, Hong Kong SAR, China; 2 School of Medicine and School of Biosciences, University of Birmingham, Birmingham, UK; 3 Research Centre of Heart, Brain, Hormone and Healthy Aging (HBHA), University of Hong Kong, Pokfulam, Hong Kong SAR, China

**Keywords:** uncoupling proteins, mitochondria, Parkinson's disease, ATP, oxidative stress, neuroprotection

## Abstract

This review gives a brief insight into the role of mitochondrial dysfunction and oxidative stress in the converging pathogenic processes involved in Parkinson's disease (PD). Mitochondria provide cellular energy in the form of ATP via oxidative phosphorylation, but as an integral part of this process, superoxides and other reactive oxygen species are also produced. Excessive free radical production contributes to oxidative stress. Cells have evolved to handle such stress via various endogenous anti-oxidant proteins. One such family of proteins is the mitochondrial uncoupling proteins (UCPs), which are anion carriers located in the mitochondrial inner membrane. There are five known homologues (UCP1 to 5), of which UCP4 and 5 are predominantly expressed in neural cells. In a series of previous publications, we have shown how these neuronal UCPs respond to 1-methyl-4-phenylpyridinium (MPP^+^; toxic metabolite of MPTP) and dopamine-induced toxicity to alleviate neuronal cell death by preserving ATP levels and mitochondrial membrane potential, and reducing oxidative stress. We also showed how their expression can be influenced by nuclear factor kappa-B (NF-κB) signaling pathway specifically in UCP4. Furthermore, we previously reported an interesting link between PD and metabolic processes through the protective effects of leptin (hormone produced by adipocytes) acting via UCP2 against MPP^+^-induced toxicity. There is increasing evidence that these endogenous neuronal UCPs can play a vital role to protect neurons against various pathogenic stresses including those associated with PD. Their expression, which can be induced, may well be a potential therapeutic target for various drugs to alleviate the harmful effects of pathogenic processes in PD and hence modify the progression of this disease.

## Review

### Mitochondrial dysfunction, oxidative stress and Parkinson's disease

Parkinson's disease (PD) is a common neurodegenerative disorder and increasingly a major burden in an aging population. Although its pathogenesis is unknown, there is evidence to implicate common pathogenic processes towards eventual cell death in PD. These processes include mitochondrial dysfunction, oxidative stress, neuroinflammation, excitotoxicity, and ubiquitin proteasome dysfunction [1-4].

There is considerable evidence to link mitochondrial dysfunction and PD. Mitochondrial Complex I activity is reduced in substantia nigra in PD [5]. Inhibition of Complex I activity using 1-methyl-4-phenyl-1,2,3,6-tetrahydropyridine (MPTP) or rotenone (both toxins used in experimental parkinsonian models) produce nigrostriatal dopaminergic degeneration in animal models [6,7]. Cybrid cell lines with normal nuclear genome but with mitochondrial DNA from PD patients have reduced Complex I activity and mitochondrial energy-dependent activities [8], have abnormal mitochondrial morphology [9], and are more susceptible to MPTP-induced toxicity. The process of aging involves the mitochondria [10]. Furthermore, dopamine metabolism and mitochondrial dysfunction generate oxidative stress. High basal levels of oxidative stress in substantia nigra are found in normal brain, and are increased in PD. Furthermore, antioxidant activity, such as glutathione (GSH), is reduced in substantia nigra of PD patients [11,12]. Based on the hypothesis that various genetic and environmental etiological factors converge on these common pathogenic processes in PD, targeting proteins which modulate mitochondria bioenergetics appears to be a logical approach in preserving neurons against mitochondrial dysfunction in PD.

### Mitochondria and ATP synthesis

Mitochondria are rod-shaped cellular organelles, which range in size from between 1 and 10 microns in length. They provide cellular energy by converting oxygen and nutrients into adenosine triphosphate (ATP) via oxidative phosphorylation. Human cells have hundreds to thousands of mitochondria per cell depending on their energy requirements [13]. Metabolically active tissues, such as neurons and red skeletal muscles, can contain over a thousand mitochondria, whereas less active tissues, such as cartilage, contain only a few hundred. Mitochondria numbers can also vary within the same cell by fission or fusion, depending on energy requirements at a specific time period. Two specialized membranes ensemble a mitochondrion namely the mitochondrial inner and outer membranes. The inner membrane is highly convoluted to make up the cristae. It also contains a group of proteins which form the electron transport chain (ETC). Oxidation of biofuels (e.g. glucose) in the Krebs cycle supplies high-energy electrons in the form of NADH or FADH_2 _to undergo oxidative phosphorylation which involves the flow of these high-energy electrons along the ETC, from Complex I and Complex II to Complex IV to molecular oxygen. Along with the flow of electrons through the ETC, there is a concomitant pumping of protons in Complex I, III, and IV from the mitochondrial matrix to the mitochondrial intermembrane space creating a proton gradient (mitochondrial membrane potential; MMP) across the inner membrane [14]. Complex V (ATP synthase) utilizes this proton gradient to drive ADP phosphorylation and generate ATP by channeling the protons back to the matrix [15]. During the process of oxidative phosphorylation, some unpaired electrons are diverted from the ETC to interact with molecular oxygen and form reactive superoxides as harmful byproducts. These ions readily interconvert to other reactive oxygen species (ROS), e.g., hydroxyl ions and H_2_O_2_, causing oxidative stress. Therefore, mitochondrial ROS generation and ATP synthesis are inevitable linked.

### Uncoupling proteins

**Figure 1:**
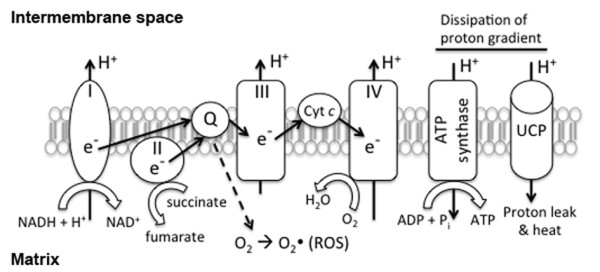
**Simplified diagram of oxidative phosphorylation and involvement of uncoupling proteins (UCPs) in mitochondrial uncoupling**. UCPs act as an alternative route to dissipate proton gradient across the inner membrane and bypass ATP synthase. Through mitochondrial uncoupling, UCPs help to minimize ROS generation caused by interactions between leaking electrons (e^-^) and molecular oxygen (O_2_).

Uncoupling proteins (UCPs) belong to a distinctive superfamily of mitochondrial transporters that uncouple biofuel oxidation from ATP synthesis by providing an alternative route to partially dissipate the mitochondrial membrane potential across the inner membrane in form of heat [16,17] (Figure 1). UCP activity has been proposed as a protective mechanism to minimize ROS generation during oxidative phosphorylation by dissipation of hyperpolarized MMP, termed as "*mild uncoupling*" [18,19]. Such slight dissipation of membrane potential has been proposed to reduce the formation of ROS without significant effects on ATP synthesis [20]. Uncoupling protein-1 (UCP1) was initially identified from brown adipose tissues (BAT) [21]. It functions to regulate energy expenditure via uncoupling biofuel oxidation from ATP synthesis and dissipates the proton gradient in the form of heat, contributing to the "non-shivering" thermogenesis in mammals. UCP1 (or thermogenin) is a key protein in maintaining body temperature in hibernating animals. However, the physiological significance of UCP1 in human is unclear because human adults possess little if any BAT [22]. At least four other structural homologues (UCP2-5) have been identified in different mammalian tissues [16]. Unlike UCP1, UCP2 is ubiquitously expressed at varying levels in different tissues including brain [23], and it is probably the most extensively studied UCP homologue so far. UCP3 is expressed in skeletal muscles and heart [24]. UCP4 and UCP5 are predominantly expressed in neural tissues [25,26], although their mRNAs are also expressed at lower levels in other peripheral tissues, such as heart, lung, and kidney [27-29]. These UCP homologues form a subfamily of mitochondrial anion carriers [30,31] distinct from other anion carriers such as ATP/ADP carriers (ANT) and phosphate carriers. These homologues have different expression levels and responses against oxidative stress among various tissues [32-35].

### Overexpression versus knockdown studies in neuronal UCPs

There has been some concern over the validity of functional studies using cells that overexpress neuronal UCPs in that overexpression may cause misfolded protein accumulation in yeast mitochondria, leading to artefactual observations [22]. Whilst we cannot exclude such a possibility, we think it is unlikely as misfolded proteins generally tend to result in abnormal cells with poorer function against cellular stresses. In contrast, we found that UCP4 and UCP5 overexpression resulted in healthier neural cells with faster cellular proliferation, better preservation of cellular ATP levels, and lower oxidative stress under MPP^+^- and dopamine-induced toxicity [36,37]. Furthermore, UCP4 and UCP5 overexpressed proteins were clearly confined to the mitochondrial fraction, and did not extend to the cytosolic fraction in cell lysates. In electron microscopy of neural cells which overexpressed UCP4, we observed normal mitochondrial morphology with intact inner membrane and cristae (Philip WL Ho: *Uncoupling protein-4 (UCP4) increases mitochondrial ATP supply by respiratory Complex II activation in neuronal cells*, submitted). Nevertheless, studies using knockdown or overexpression cellular systems are not mutually exclusive, and it would be ideal if both sets of systems are tested concurrently.

### Functional properties of neuronal UCPs

#### 1) Thermogenesis and neuronal plasticity

**Figure 2:**
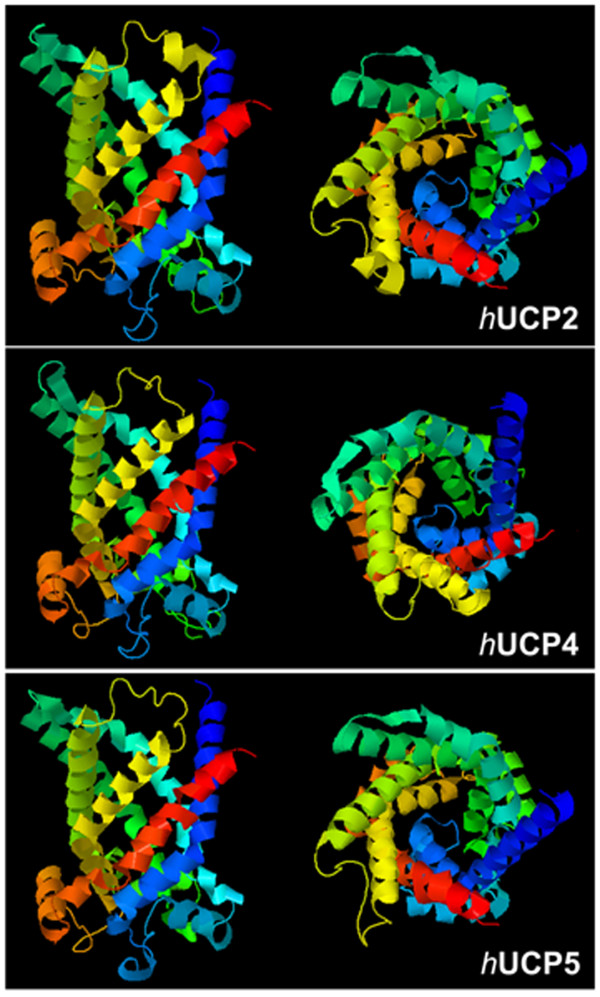
**Computer prediction of tertiary structures of human UCP2, UCP4, and UCP5 proteins**. These homologues share a similar six trans-membrane channel-like structure despite having significant differences in their amino acid identities.

Among the five homologues, UCP2, UCP4 and UCP5 (neuronal UCPs) are found in neural tissues, and they will be collectively termed as neuronal UCPs for the purposes of this review. They share a similar six trans-membrane tertiary structure (Figure 2) indicating similar channel-like functions despite having significant differences in their amino acid identity [25,26,28]. The physiological significance of neuronal UCPs in human is unclear, particularly for UCP4 and UCP5. It is unclear whether these homologues work synergistically in neuronal system, and whether there is some degree of functional redundancy evolved from a common ancestral gene [38]. Although they are predominantly expressed in neural tissues, the link between their uncoupling activities, neuronal function, and plasticity is unclear. Based on micro-regional temperature changes in mouse brain, it was suggested that UCP2 expression may regulate thermogenesis via mitochondrial uncoupling in the microenvironment, where the resultant elevated temperature facilitates chemical diffusion and neural transmission in synapses [39,40]. Compared to UCP2, UCP4 and UCP5 are expressed in neural tissues at a much higher level by at least one order of magnitude [29,41]. It is not surprisingly that UCP4 and UCP5 may well exert a more profound regulatory role than UCP2 in determining neuronal plasticity and survival.

#### 2) Neuroprotection against oxidative stress

Although major anti-oxidative defenses such as superoxide dismutase, glutathione, and catalase in mitochondria can reduce harmful effects of superoxides, there is increasing evidence that neuronal UCPs can also play an important role to protect against oxidative stress from their specific location in mitochondria and uncoupling properties. The "*mild uncoupling" *hypothesis has been proposed to explain the protective mechanisms of how UCPs can decrease ROS generation in mitochondria [18,19]. (Figure 1).

There is evidence to show the neuroprotective properties of UCPs. UCP2 expression was critical in reducing ROS generation in brain of UCP2-knockout mice, and mice that overexpress human UCP2 have lower dopaminergic cell loss against MPTP toxicity [42]. UCP2 can reduce mitochondrial ROS by facilitating fatty acid hydroperoxides cycling and proton leak [43]. Superoxides generated during respiration can induce lipid peroxidation, which in turn activates UCPs to increase proton leak to diminish superoxide production in a negative feedback loop [44]. Neuronal UCP expression appears to be responsive to oxidative stress in various *in vitro *and *in vivo *experimental models of PD [32-34]. UCP2 and UCP5 expression were up-regulated in brains of patients after developing ischemic lesions from embolic stroke and multiple infarction [33]. Similar induction of UCP2 and UCP5 expression was also observed in colonic cells under oxidative stress, demonstrating a potential local feedback mechanism in counteracting oxidative damage and mitochondrial dysfunction [35]. We observed a time- and dose-dependent induction of UCP2, 4, and 5 expression in human neuronal cells after exposure to MPP^+^, the toxic metabolite of MPTP [32]. MPP^+ ^specifically inhibits mitochondrial Complex I activity, which impairs oxidative phosphorylation and subsequently causes ATP deficiency and oxidative stress. Neuronal UCP mRNA expression increased with increased MPP^+^-induced toxicity. We postulated that these increases in their gene expression served to protect the neurons against MPP^+ ^toxicity. To explore this hypothesis, we knocked down UCP5 expression by siRNA in SH-SY5Y neuronal cells and found that reduced UCP5 expression exacerbated MPP^+^-induced mitochondrial depolarization and induced apoptosis, indicating that UCP5 played a significant role in protecting the neurons against MPP^+^-induced toxicity [45]. This finding was supported by our later study where we stably overexpressed UCP5 expression in these cells, and demonstrated its protective properties [37]. We found that overexpressing UCP5 could preserve MMP and ATP levels, and suppress oxidative stress induced by MPP^+^. Similarly, we demonstrated the neuroprotective properties of UCP4 using SH-SY5Y neuronal cells overexpressing UCP4 under MPP^+ ^toxicity. We found that increasing UCP4 expression could preserve cellular ATP levels and MMP, which made these neuronal cells more resistant to MPP^+^-induced ATP deficiency and oxidative stress. Furthermore, it is interesting to note that UCP2 expression in response to MPP^+^-induced mitochondrial dysfunction could be effectively suppressed by overexpressing UCP4, indicating a functional link between UCP2 and UCP4 [36].

#### 3) Regulation of mitochondrial membrane potential and ATP level

Neurons require considerable energy for their activities, including synaptic neurotransmission, and hence have significant numbers of mitochondria, especially at their synaptic nerve terminals. Oxidative phosphorylation in mitochondria plays a major role to supply neurons with ATP. Unlike other cell types that are able to utilize glycolysis as an alternative energy source, glycolysis in fully differentiated neurons is intrinsically suppressed to maintain their antioxidant status [46]. This property makes neurons highly vulnerable to ATP deficiency, and may be a factor in the susceptibility of nigrostriatal dopaminergic neurons to cell death in PD where their major energy supply via mitochondrial Complex I is impaired [47].

The "*mild uncoupling*" hypothesis postulates that the UCP-mediated proton leak from the intermembrane space to the mitochondrial matrix across the inner mitochondrial membrane, which results in mild uncoupling, reduces the harmful effects of excessive ROS generation at the expense of ATP production. Although this indicates that the amounts of proton leak should parallel levels of MMP in the uncoupling process, there is as yet little definite evidence that UCP expression can directly affect cellular ATP levels. There is evidence that changes in MMP may not necessarily correlate to overall ATP levels. Knockdown of UCP2 and 3 in human epithelial cells did not affect either MMP or ATP levels [48]. We found that SH-SY5Y neuronal cells with stably knocked down UCP2 expression showed higher MMP but decreased cellular ATP levels [49]. Although UCP2 overexpression in mouse liver cells was reported to decrease ATP levels [50], other groups reported markedly higher ATP levels in the hippocampus of UCP2 transgenic mouse [51]. It is unclear whether changes in ATP levels is a secondary effect from dissipation of MMP by UCPs, or a result of direct UCP interaction with other factors such as the ATP synthesis machinery or mitochondrial biogenesis. Furthermore, there are also other ion carriers in mitochondria that can affect MMP, such as ADP/ATP translocase (ANT). Hence, the role of neuronal UCPs and their effects on MMP and ATP levels may be much more complex than it appears. Nevertheless, we observed that neuronal cells overexpressing UCP4 showed a significantly higher level of cellular ATP compared to those cells expressing endogenous levels of UCP4 [36]. To explain such increase in ATP level, we recently discovered that UCP4 overexpression resulted in increased mitochondrial oxygen consumption through interacting with respiratory Complex II to promote ATP synthesis (Philip WL Ho: *Uncoupling protein-4 (UCP4) increases mitochondrial ATP supply by respiratory Complex II activation in neuronal cells*, submitted), in keeping with a recent study where UCP4 was shown to increase succinate transport via Complex II in *C. elegans *[52]. UCP4 overexpression in rat PC12 adrenal pheochromocytoma cells induced glucose uptake and shifted the mode of ATP synthesis from oxidative phosphorylation to glycolysis to maintain overall ATP supply [53]. Although the role of glycolysis in maintaining overall ATP supply in fully differentiated neurons is still unclear, the evidence so far indicates an important role for UCP4 in maintaining cellular energy supply to protect neuronal cells against ATP deficiency. Unlike UCP4, UCP5 overexpression resulted in lower ATP levels under normal culture conditions. It appears that UCP2 and UCP5 had "typical" uncoupling properties unlike UCP4. It is unclear why these neuronal UCP homologues had such a divergent effect on ATP levels even though they all showed neuroprotective properties under MPP^+^-induced oxidative stress and ATP deficiency. Although they are all evolved from a common ancestral gene [54], we postulate that this functional difference in neuronal UCPs sited at the inner mitochondrial membrane may serve to better protect the cell from various forms of cellular stresses by providing a possible alternative mechanistic route on protection. Even accounting for functional redundancy, there is no reason why neuronal UCPs sited in the same location of the mitochondria need to act in exactly the same manner to protect the cell from cellular stresses. It has been postulated that dissimilarity of UCP4 from the other UCP homologues may be due to structural differences in that UCP4 exhibits a distinctive helical profile when associated with negatively charged phospholipid vesicles and shows different purine nucleotide binding properties compared to other UCP homologues [55].

#### 4) Calcium homeostasis

Another important function of UCPs is the regulation of calcium homeostasis. Unlike many other types of neurons, dopaminergic neurons in substantia nigra are autonomously active. The L-type Ca^2+ ^channels during autonomous pacemaking were shown to sensitize dopaminergic neurons to toxins in PD experimental models [56]. In a study using human endothelial cells, UCP2 and UCP3 were shown to be crucial for mitochondrial Ca^2+ ^sequestration in response to cellular stresses [57]. Whether UCP2 and UCP3 play a similar role in dopaminergic neurons is unclear. UCP4 overexpression in neural cells stabilized Ca^2+ ^homeostasis in response to thapsigargin-induced endoplasmic reticulum Ca^2+ ^store depletion, preserved mitochondrial function, reduced mitochondrial ROS generation, and increased cell survival against oxidative stress [58]. UCP4 knockdown in primary hippocampal neurons resulted in calcium overload and cell death [53]. Superoxides can affect mitochondrial free Ca^2+ ^by regulating UCP expression in neuronal cells [59]. It is interesting to note that mice with knock out of DJ-1 (mutations of which has been associated with an autosomal recessive young onset form of PD) had reduced UCP4 and 5 expression specific to the substantia nigra pars compacta, sparing the cortex, hippocampus and the ventral tegmental area, indicating that these neuronal UCPs may play a role in calcium-induced uncoupling specifically in SNc DA neurons [60].

#### 5) Link between metabolism and neuroprotective effects of leptin: role for UCP2

Metabolic pathways have been linked to aging and neurodegenerative processes. Metabolic intervention can prolong lifespan, decrease the incidence of age-related diseases, improve stress responses, and maintain physiological function in experimental and epidemiological studies [61-63]. Metabolic intervention, e.g. low-calorie diet, can promote survival of dopaminergic neurons in a primate model of PD by amelioration of neurochemical and motor deficits [64]. Leptin, a hormone produced by adipocytes [65], regulates basal metabolism by modulating neuropeptides in hypothalamus [66], where its levels are affected by glucose levels and fasting [67-71]. Leptin acts on an array of signaling pathways by specific binding to leptin receptors (ObR), which are widely expressed in brain, including DA neurons [72]. Leptin has anti-apoptotic properties [73]. We found that leptin protected neuronal cells against mitochondrial dysfunction induced by MPP^+ ^by inducing UCP2 expression to preserve MMP and ATP levels [49]. Such protective effects were abolished by knocking down UCP2 expression using siRNA, indicating that UCP2 mediates leptin protection against MPP^+ ^toxicity to promote neuronal cell survival by preserving mitochondrial function [49]. One possible reason to explain why leptin could preserve cellular ATP levels under MPP^+^-induced ATP deficiency may be its well-known function in activating AMPK and regulating cellular energy homeostasis [74]. Furthermore, leptin can induce an insulin-like signaling pathway involving PI3K-dependent activation of PDE3B (phosphodiesterase 3B) which reduces cAMP in the central nervous system. Because multiple cAMP-response elements have been identified in the promoter region of human UCP2 [75], and its expression is stimulated by the cAMP/PKA signal cascade [76], modulation of UCP2 expression by leptin may well be mediated via cAMP signaling. Leptin can enter the brain [77], and directly acts on neurons, including dopaminergic neurons [78]. Obese (ob/ob) mice, which lack functional leptin, have increased "proton leak" compared with lean controls, which demonstrates beneficial effects of leptin to mitochondrial function [79].

### Gene regulation of neuronal UCPs

**Figure 3:**
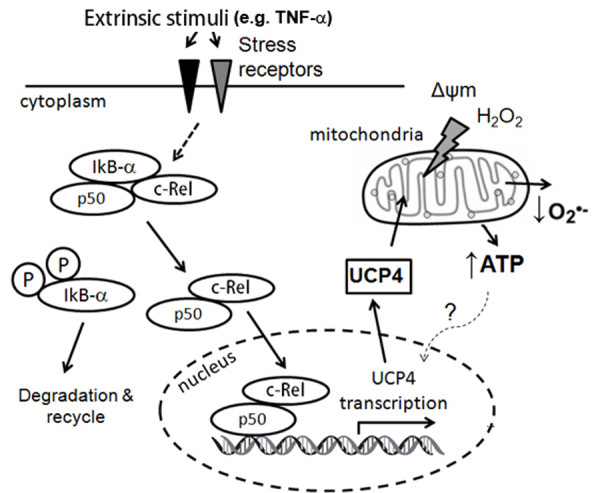
**Schematic diagram showing proposed neuroprotective properties of UCP4 against oxidative stress in the pro-survival NF-κB pathway**. External stimuli (e.g. TNF-α) activate NF-κB subunits (e.g. c-Rel, p50) via phosphorylation of IκB-α. Nuclear translocation of c-Rel and p50 promote transcription of UCP4 gene. Increased expression of UCP4 in mitochondria suppresses oxidative stress by stabilizing mitochondrial membrane potential and preserving cellular ATP level.

Nuclear factor kappa-B (NF-κB) is a heterodimeric transcription factor that translocates to the nucleus and mediates the transcription of proteins involved in cell survival and proliferation, inflammatory response, and anti-apoptotic factors in neurons, astrocytes, microglia, and oligodendrocytes [80-83], including defense against oxidative stress. NF-κB is crucial in regulating neuronal survival by specific activation of diverse NF-κB complexes [84,85], which are composed of five different subunits: RelA (p65), c-Rel, RelB, p50 (NF-κB1), and p52 (NF-κB2). A close association of NF-κB activation exists with the neuropathology found in neurodegenerative processes in PD [85-87]. Activation of NF-κB has been reported in the substantia nigra of mice treated with MPTP [88,89]. The subunit p50/c-Rel can activate several pro-survival genes, such as bcl-2 and Mn-SOD [90,91]. Within 2 kb of the 5'-flanking region upstream of the transcription start site of human UCP4 gene, we identified a functional NF-κB binding site in the promoter region. UCP4 promoter activity and gene transcription were activated via this binding site after exposure to TNF-α (tumor necrosis factor) and MPP^+ ^[92]. TNF-α is a cytokine involved in neuroinflammation and an activator of NF-κB pathways involved in the cellular stress response [93,94]. Based on the transcriptional response of UCP4 gene expression by TNF-α and its protective effects against ATP deficiency and oxidative stress induced by MPP^+ ^in neuronal cells, we proposed a functional role for UCP4 to mitigate against mitochondria dysfunction and oxidative stress via p50/c-Rel-mediated NF-κB pathway (Figure 3).

UCP5 was named brain mitochondrial carrier protein (BMCP1) because of its partial homology with other uncoupling proteins. The promoter region of human UCP5 gene has not been fully characterized. However, transcription of this gene gives rise to three alternative spliced mRNA products, termed long, short, and short with insert respectively [26,27]. Our search of the 5'-flanking region of human UCP5 gene revealed four cAMP response elements 5 kb upstream of the transcription start site [95]. Our initial studies in treating SH-SY5Y cells with dibutrylyl-cAMP indicated that UCP5 mRNA (short form) exhibited a dose-dependent increase after treatment. cAMP is an important secondary messager in intracellular signal transduction of a wide variety of biological processes associated with protein kinase A (PKA). The significance of the transcriptional response of UCP5 to cAMP awaits further investigation.

Sirtuin 1 (Sirt1) is a protein which can modulate cell senescence and longevity. It is an important repressor of UCP2 gene transcription by binding to the UCP2 promoter region, and modulates the amount of insulin secretion in pancreatic β-cells [96]. Sirt1 can also affect Agrp neuronal firing and synaptic plasticity via UCP2 [97]. UCP2 may well play a role in modulating mitochondrial energy homeostasis downstream of a Sirt1-mediated regulatory cascade of cell senescence in brain. UCP2 expression can be up-regulated by preconditioning in hippocampus [98], suggesting a role of UCP2 in anti-oxidative protection against ischemic/reperfusion injuries.

### Therapeutic implications in Parkinson's disease

Current treatment of PD does not address the underlying dopaminergic nigrostriatal neurodegeneration, or alleviate the progressive motor or non-motor disability associated with degeneration of either dopaminergic or non-dopaminergic pathways. Hence, treatment strategies that can modify the progressive course of PD and delay its progression should be developed to address this unmet need. One such strategy involves alleviating the harmful effects of downstream pathogenic processes by targeting mitochondrial dysfunction and oxidative stress in PD. Neuronal UCPs possess properties that can protect neuronal cells against various cellular stresses, including stresses observed in experimental parkinsonian models. Furthermore, the expression of these UCPs is inducible. Compounds that can induce endogenous neuronal UCP expression can be developed into potential therapies in PD. We have shown that UCP4 expression can be induced by activators of NF-κB signaling pathway. In the central nervous system, NF-κB is an important nuclear transcription factor in regulating neurodegenerative pathways [99,100], and it plays a crucial role in determining neuronal survival and neuroplasticity [101-103]. Although the relationship between NF-κB activation and the pathogenesis PD is unclear, there is some evidence to link NF-κB activation with potential disease-modifying effects in PD. There was an increase in IκBα expression and inhibition of translocation of the p65 NF-κB subunit to the nuclei of dopaminergic neurons, glial cells and astrocytes; effects which were correlated with the protective effects of pioglitazone in exerting anti-inflammatory effects in mice exposed to MPTP-induced toxicity [104]. In post-mortem studies, NF-κB expression was increased in substantia nigra of PD patients [105,106]. Furthermore, *in vitro *studies showed activation of NF-κB in response to 6-hydroxydopamine toxicity [107]. The link between NF-κB and UCP4 may provide a possible therapeutic strategy to preserve the function of affected neurons in PD, by for example, inducing UCP4 expression using NF-κB c-Rel activators, and alleviating mitochondrial dysfunction and oxidative stress.

Apart from PD, UCP4 variants have been linked to other neurological disorders such as multiple sclerosis [108], schizophrenia [109,110], leukoaraiosis [111] and Alzheimer's disease [112]. Decreased UCP2, 4, 5 expression impaired the ability of neurons from brain affected by Alzheimer's disease brain to be protected from oxidative damage [112]. Increasing evidence indicates that neuronal UCPs may well play a crucial role in neuronal survival when they are under stress. Neuronal UCPs may be a potential therapeutic target for the treatment of these neurological disorders.

## Competing interests

The authors declare that they have no competing interests.

## Authors' contributions

WL, WM, and HF Liu carried out the molecular and functional studies. HF So and HM carried out the cell culture, sample preparation and extraction. WL, DB, KH and SL participated in the design of studies. WL and DB performed the statistical analyses. WL, DB, WM and SL helped to draft the manuscript. All authors read and approved the final manuscript.
